# Impact of Ramadan Fasting on Mental Health, Body Composition, Physical Activity, and Sleep Outcomes Among University Students

**DOI:** 10.3390/healthcare13060639

**Published:** 2025-03-14

**Authors:** Nour Amin Elsahoryi, Mohammed O. Ibrahim, Omar Amin Alhaj, Gaida Abu Doleh, Abeer Ali Aljahdali

**Affiliations:** 1 Department of Nutrition, Faculty of Pharmacy and Medical Sciences, University of Petra, Amman 11196, Jordan; omar.alhaj@uop.edu.jo (O.A.A.);; 2 Department of Nutrition and Food Technology, Faculty of Agriculture, Mutah University, Karak 61710, Jordan; mohammedomar@mutah.edu.jo; 3 Department of Clinical Nutrition, Faculty of Applied Medical Sciences, King Abdulaziz University, Jeddah 21589, Saudi Arabia; aaoaljahdali1@kau.edu.sa; 4 Department of Nutritional Sciences, School of Public Health, University of Michigan, Ann Arbor, MI 48109, USA

**Keywords:** Ramadan fasting, mental health, sleep quality, body composition, university students

## Abstract

Background: Over two billion Muslims across the world practice Ramadan fasting, which involves refraining from food and drink from dawn to sunset. This study was conducted to investigate the effects of Ramadan fasting on mental health (depression, anxiety, stress), body composition, physical activity, and sleep quality among Jordanian university students. Methods: This study utilized a quasi-experimental, pre–post-intervention design. It was conducted between March and April 2024 and included 77 students from the University of Petra (UOP) in Amman, Jordan. Bivariate analysis was performed to compare the variables pre–post Ramadan fasting. A linear mixed-effects model assessed the association between Ramadan fasting and each outcome. Results: The results indicated that Ramadan fasting was not associated with a change in the Depression Anxiety Stress Scales-21 (DASS-21 score). Ramadan fasting led to a decrease in sleep quality, indicated by an increase in the PSQI score (β = 0.92; *p*-value = 0.0419). Component analysis revealed statistically significant changes in subjective sleep quality (*p*-value = 0.0009), sleep duration (*p*-value < 0.0001), and sleep disturbances (*p*-value = 0.025). Body composition: Ramadan fasting was significantly associated with a decrease in the number of fat components, such as weight (β = −1.20; *p*-value = 0.0116), body mass index (β = −0.55), waist circumference (β = −1.79; *p*-value = 0.0029), fat mass index (β = −0.43; *p*-value = 0.0279), visceral fat area (β = −6.86; *p*-value = 0.0383), and body adiposity index (β = −0.45; *p*-value = 0.0068) (all *p*-value < 0.05). No substantial alterations to the body’s water-related properties were noted. Physical Activity: A marked decrease was noted in moderate- to high-intensity activity levels (*p*-value < 0.0001). Conclusion: In conclusion, Ramadan fasting can positively affect body composition through a reduction in weight, body mass index, waist circumference, and other parameters of fat, emphasizing its potential role in body composition improvement. However, fasting was also accompanied by poorer sleep quality, including, specifically, poorer subjective sleep quality and sleep duration and greater disturbance. The associated impairments to sleep revealed in these findings demand strategies to mitigate sleep impairments, alongside, where possible, potential beneficial effects of fasting on body composition.

## 1. Introduction

Ramadan fasting is practiced by **over** 1.9 billion Muslims worldwide and involves abstaining from food, drink, smoking, and other behaviors from dawn to sunset throughout the holy month [1,2]. Beyond its religious significance, fasting is associated with various physiological and psychological benefits, influencing metabolism, mental health, and overall well-being. Many studies highlight the positive effects of Ramadan fasting, including enhanced self-discipline, emotional resilience, and spiritual fulfillment [3]. Additionally, the communal aspects of Ramadan, such as shared meals, nightly prayers, and charitable acts, promote social bonding and reduce feelings of loneliness [4,5]. From a physiological standpoint, fasting has been linked to improvements in body composition, metabolic regulation, and cardiovascular health. Some studies report reductions in body fat and body mass index (BMI), indicating potential metabolic benefits [6]. A systematic review and meta-analysis also found that Ramadan fasting reduced anxiety and depression levels without significantly increasing fatigue [7]. Furthermore, religious fasting encourages health-conscious behaviors, such as mindful eating and increased self-awareness, which may contribute to long-term well-being and sustainability [8].

However, despite these potential benefits, Ramadan fasting also presents physiological and psychological challenges, particularly for university students who must balance academic responsibilities, social commitments, and fluctuating energy levels [8,9]. Disruptions in sleep patterns, hydration levels, and meal timing can contribute to fatigue, mood fluctuations, and cognitive difficulties, all of which may negatively impact academic performance [10]. Sleep deprivation, combined with prolonged fasting hours, may also reduce concentration, alter mood stability, and decrease physical activity levels [11,12]. Beyond physical strain, some students experience psychological stress due to societal and cultural expectations surrounding fasting. The pressure to fast, whether from family, religious beliefs, or peer influence, can create mental distress—especially for students who may be medically exempt or struggle with fasting due to personal reasons [13,14,15]. The challenge is even greater for Muslim students studying in non-Muslim-majority countries, where institutional schedules do not accommodate fasting routines, making it harder to manage academic performance. Hessel Oosterbeek and Bas van der Klaauw (2013) found that Ramadan, which shifts yearly, negatively affects Muslim students’ academic performance in non-Muslim countries, further emphasizing the need for supportive academic policies during this period [16]. These psychosocial pressures highlight the need for effective support strategies to help students navigate Ramadan while maintaining their well-being.

Despite extensive research on Ramadan fasting, studies examining its comprehensive impact on mental health, sleep quality, and body composition among university students—particularly in the Middle East—remain limited. Most research has analyzed these factors individually, but an integrated assessment considering their interplay is lacking. Understanding how fasting affects these aspects holistically is essential for developing better fasting management strategies, ensuring that students maintain their academic performance and their physical and mental health. Therefore, this study aims to investigate the effects of Ramadan fasting on mental health, body composition, physical activity, and sleep quality among university students in Jordan using various adjusted models. By addressing both the benefits and challenges of fasting, this research seeks to contribute to improved fasting management strategies, helping students optimize their well-being, sustain their academic performance, and mitigate potential health risks during Ramadan.

## 2. Materials and Methods

### 2.1. Research Design

This study utilized a quasi-experimental, pre–post-intervention design, which is appropriate for evaluating changes over time within the same population. The study was conducted between March and April 2024 at the University of Petra (UOP) in Amman, Jordan. The protocol and consent method adhered to the latest revised Declaration of Helsinki (2013) and received approval from the Human Research Ethics Committee of the University of Petra (approval number: S/6/7/2024). Written informed consent was obtained from each student before they participated in the study.

### 2.2. Participants and Setting

A total of 77 students voluntarily participated and completed the study. The research was conducted at the Nutrition Center in the Pharmacy and Medical Sciences Department, UOP, Amman, Jordan. The recruitment process included electronic leaflets on the university’s website and a snowball sampling strategy via social media and faculty networks. While this method effectively reached a broad student demographic, it may have introduced selection bias, as participation was voluntary. To mitigate this, a large-scale awareness campaign was conducted through university platforms to ensure diverse representation. Eligibility criteria included students aged 18–35 years, students fasting during Ramadan, and non-smokers. Exclusion criteria comprised students with acute or chronic diseases, medication use, specific dietary restrictions, pregnancy, breastfeeding, contraceptive use, those who started fasting one week before Ramadan, students who fasted less than 15 days during the month, and those with a BMI over 40 or under 20. While these BMI cutoffs ensured a homogeneous sample, they may limit the generalizability of findings to individuals outside of this range. A sample size calculation was performed to determine the required number of participants. Based on previous quasi-experimental studies, a minimum of 40 participants was needed to achieve 80% power with a *p*-value of 0.05 to detect a significant 1 kg difference in BMI before and after fasting. This estimate was derived from prior research showing an expected effect size of 0.20 [17].

### 2.3. Data Collection

Baseline data were collected three days before Ramadan fasting at the Nutrition Research Center (University of Petra), with follow-up data collected immediately after the fasting period (29 days). To ensure consistency, all measurements were taken at the same time of day (morning, fasting state) to minimize diurnal variations. This year, Muslims in Amman, Jordan fasted for an average of approximately 15 h and 13 min per day based on the calculation methods of Muslim prayers. Participants were advised not to change their dietary intake, exercise, or social habits during the study period to maintain internal validity. Sociodemographic and medical history data were obtained via an online questionnaire and subsequently screened by the research investigators, followed by body composition assessments. The research team collected the data and conducted the measurements following consensus on the work and data collection protocol. Three validated questionnaires were administered pre- and post-Ramadan to assess mental health, sleep quality, and physical activity levels. Appointments were arranged for the students two days before they began fasting, allowing the research team to take body composition measurements and for the students to complete the questionnaires (self-reporting) on the same day. This protocol was repeated at the end of the Ramadan fasting month, which marked the study’s endpoint.

### 2.4. Measures

The questionnaires included assessments of depression, anxiety, and stress using the DASS 21 scale [15,18,19], sleep quality using the Pittsburgh Sleep Quality Index (PSQI) [20], and physical activity levels using the General Practice Physical Activity Questionnaire (GPPAQ) [21].

DASS 21 is a validated, reliable, and high-quality tool for evaluating depression, anxiety, and stress, as demonstrated in previous scientific articles and a recent systematic review [22]. The version used in the current study [23] consists of 21 questions, each evaluated on a four-point scale ranging from “Did not apply to me at all” to “Applied to me very much, or most of the time”. The total score for each scale is multiplied by 2, with higher scores indicating higher levels of disorder. The scores can be classified categorically also, as normal (0–9), mild (10–13), moderate (14–20), severe (21–27), and extremely severe (≥28).

The PSQI contains a mix of open-ended and Likert-type questions, which are converted into a digital score according to the classification by the main author [20]. Each question is scored from 0 to 3, with higher values indicating greater difficulties. The total score ranges from 0 to 21, with higher scores representing poor sleep quality and greater sleep difficulties.

The GPPAQ is a reliable and validated tool for assessing the physical activity level (PAL) of adults [21]. It consists of seven questions and takes about a minute to complete. The final score classifies individuals as sedentary, moderate, active, or very active. More detailed scoring information for each questionnaire can be found in the original published research.

Overall, the questionnaires take less than 10 min to complete, and it does not require specific personal skills to complete. The Arabic versions were used, as all participants are fluent in English and study scientific specializations in the English language.

Body composition was assessed using a Body Composition Analyzer (BIA devices, 970. Model 1421000139). Measurements were taken twice—before and after Ramadan—in a controlled setting, ensuring participants were fasted, properly hydrated, and wearing light clothing to standardize conditions [24]. The study adopted the following measurements: BMI, weight in kilograms divided by height in square meters, basal metabolic rate (BMR, kcal), fat percentage, fat mass (FM, kg), fat-free mass (FFM, kg), lean mass (kg), total body water (TBW, L), extracellular water (ECW, L), intracellular water (ICW, L), visceral fat area (VFA, cm^2^), and visceral adipose tissue (g), which explain the nutritional and hydration status of the participants. To minimize measurement variability, the study protocol accounted for menstrual cycle variations among female participants to ensure consistent hydration and body composition status at both time points.

### 2.5. Statistical Analysis

Descriptive statistics were reported as means ± standard deviations for continuous variables and frequencies/percentages for categorical variables. The distribution of outcomes was visually inspected using histograms and box plots to assess normality. To compare differences between pre- and post-fasting data, Wilcoxon signed-rank tests were used for continuous variables, McNemar’s test was applied to binary outcomes, and Bowker’s test of symmetry was used for categorical variables. For continuous variables, Cohen’s d effect size was calculated by standardizing the mean difference between the two study visits (T1–T2) by dividing the difference by the baseline standard deviation. Cohen’s d values were interpreted as 0.20 (small), 0.50 (medium), and 0.80 (large) effect sizes [25]. To assess the association between Ramadan fasting and each outcome, a linear mixed-effects model was employed, accounting for repeated measures. The model adjustments included an unadjusted model (Model 1), a model adjusted for age (Model 2), a model adjusted for age and sex (Model 3), and a model adjusted for age, sex, and physical activity (Model 4). To ensure the validity of these models, assumptions were inspected by checking the residuals of the final models for each outcome. Findings from the linear mixed-effects models were presented as beta estimates (β) with standard errors (SEs). Statistical analyses were conducted using SAS 9.4 (Statistical Analysis Systems software), with *p* < 0.05 considered statistically significant.

## 3. Results

Table 1 presents the baseline demographic characteristics of the study population. The mean age was 21.22 ± 2.04 years, with an age range of 18 to 28 years. Female participants accounted for 72.73% of the sample, while males made up 27.27%. The majority of participants were single (94.32%), with only 5.68% being married. Regarding smoking status, 60.23% were nonsmokers, 30.68% were current smokers, and 9.09% were ex-smokers. Most participants reported living with their families (78.41%), followed by living alone (15.91%) and in students’ housing (5.68%).

### 3.1. Mental Health Outcomes

Table 2 summarizes changes in mental health indicators before and after Ramadan fasting. The DASS-21 total score did not show a statistically significant change (*p* = 0.4080), and no significant differences were observed in individual components of depression (*p* = 0.4769), anxiety (*p* = 0.3058), or stress (*p* = 0.6161).

**Figure 1:**
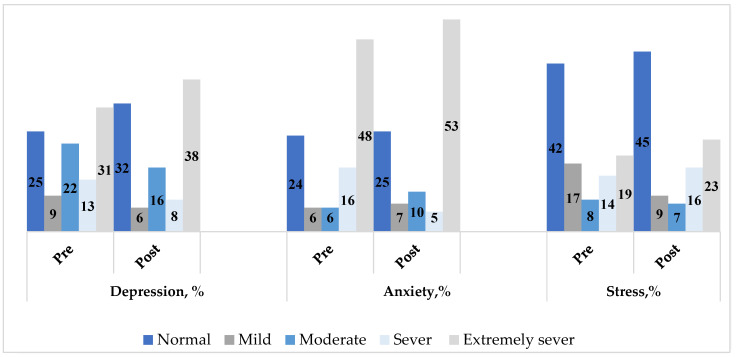
The change in depression, anxiety, and stress before and after Ramadan fasting (N = 77). *p*-value for depression= 0.7431; *p*-value for anxiety= 0.2568; *p*-value for stress= 0.9140. Bowker’s symmetry test was used.

Figure 1 illustrates the changes in the classifications of depression, anxiety, and stress before and during Ramadan fasting. Before Ramadan fasting, the depression classifications were as follows: 25% of participants were classified as having a normal depression score, 9% as mild, 22% as moderate, 13% as severe, and 31% as extremely severe. During Ramadan fasting, these classifications changed to 32% normal, 6% mild, 16% moderate, 8% severe, and 38% extremely severe. However, the change in depression distribution between the two study visits was not statistically significant (*p*-value = 0.7431). For anxiety, the classifications before and during Ramadan fasting were similar. Before fasting, 24% of participants had a normal anxiety score, 6% had mild anxiety, 6% had moderate anxiety, 16% had severe anxiety, and 48% had extremely severe anxiety. During fasting, these classifications were 25% normal, 7% mild, 10% moderate, 5% severe, and 53% extremely severe. The change in anxiety distribution between the two study visits was not statistically significant (*p*-value = 0.2568). Regarding stress, the classifications before and during Ramadan fasting also showed little change. Before fasting, 42% of participants had a normal stress score, 17% had mild stress, 8% had moderate stress, 14% had severe stress, and 19% had extremely severe stress. During fasting, these classifications were 45% normal, 9% mild, 7% moderate, 16% severe, and 23% extremely severe. The change in stress distribution between the two study visits was not statistically significant (*p*-value = 0.9140).

As shown in Table 3, physical activity levels significantly declined during Ramadan, with a shift from moderate to high activity levels (97% before fasting) to predominantly light or sedentary activity levels (63% during fasting; *p* < 0.0001). This decrease could be attributed to reduced energy availability, altered sleep patterns, and increased fatigue.

### 3.2. Sleep Quality

Table 3 shows that the mean PSQI score increased from 5.73 ± 2.40 before Ramadan fasting to 6.65 ± 3.08 during fasting, indicating a significant deterioration in sleep quality (*p*-value < 0.0001) with a large effect size according to Cohen’s d. Within the PSQI components, significant changes were observed in subjective sleep quality (*p*-value = 0.0009), sleep duration (*p*-value < 0.0001), and sleep disturbance (*p*-value = 0.0250). However, there were no significant changes in sleep latency (*p*-value = 0.2802), sleep efficiency (*p*-value = 0.6328), use of sleep medication (*p*-value = 0.6349), or daytime dysfunction (*p*-value = 0.0719).

### 3.3. Physical Activity

As shown in Table 3, physical activity levels significantly declined during Ramadan, with a shift from moderate to high activity levels (97% before fasting) to predominantly light or sedentary activity levels (63% during fasting; *p* < 0.0001). This decrease could be attributed to reduced energy availability, altered sleep patterns, and increased fatigue.

### 3.4. Body Composition Changes

Table 4 displays the pre- and post-Ramadan body composition measurements. Statistically significant reductions were observed in weight (−1.35 kg, *p* < 0.0001), BMI (−0.52 kg/m^2^, *p* < 0.0001), waist circumference (−2.08 cm, *p* < 0.0001), FM index (−0.42, *p* < 0.0001), and VFA (−7.54 cm^2^, *p* < 0.0001). However, Cohen’s d effect sizes for these changes were small (ranging from 0.07 to 0.16), indicating that although statistically significant, the practical impact of these reductions may be modest. No significant changes were observed in skeletal muscle mass (*p* = 0.0596) or TBW (*p* = 0.0703).

### 3.5. Multivariate Analysis

Table 5 presents the effect of Ramadan fasting on depression, anxiety, stress, sleep quality, and body composition using linear mixed-effect models. In the fully adjusted model, Ramadan fasting was positively associated with PSQI score (β (SE)= 0.82 (0.32), *p*-value = 0.0134). For the anthropometric measurements, Ramadan fasting was inversely associated with weight (β (SE)= −1.20 (0.46), *p*-value = 0.0116), BMI (β (SE)= −0.55 (0.17), *p*-value = 0.0017), waist circumference (β (SE)= −1.79 (0.58), *p*-value = 0.0029), waist to hip ratio (β (SE)= −0.014 (0.005), *p*-value = 0.0041), and hip circumference (β (SE)= −0.66 (0.27), *p*-value = 0.0175). These results are interpreted as reductions of 1.20 kg in body weight, 2 cm in waist circumference, and 1.5 cm in hip circumference during Ramadan fasting compared to the baseline values. For the fat-related body composition parameters, Ramadan fasting was inversely associated with the FM index (β (SE)= −0.43 (0.19), *p*-value = 0.0279), VFA (β (SE)= −6.86 (3.26), *p*-value = 0.0383), obesity degree (β (SE)= −2.48 (0.79), *p*-value = 0.0024), and body adiposity index (β (SE)= −0.45 (0.16), *p*-value = 0.0068). Lastly, for the skeletal and lean-related body composition parameters, Ramadan fasting was only positively associated with the skeletal muscle mass–VFA ratio (β (SE)= 0.02 (0.01), *p*-value = 0.0449).

## 4. Discussion

This study is important due to its objectives of exploring the predicted changes in different outcomes after fasting during the holy month of Ramadan. The axes of this research revolved around the impact of this duty on the mental health, sleep quality, and body composition of participants. The first axis was about the impact of Ramadan fasting on mental health. The findings of this study indicated that there were no significant differences in the total mean score of DASS-21 at the baseline and the end of Ramadan. The results also showed that there were no significant differences in the mean scores of individual components of DASS-21, including depression, anxiety, and stress. Moreover, there were no significant changes in the distribution into normal, mild, moderate, severe, extremely severe categories of the aforementioned components of DASS-21. Aligned with our results were the results of a study conducted by Alsowaid et al. [26], in which they found no effect of Ramadan fasting on the levels of anxiety and depression. Similarly, Furuncuoglu et al. [27] documented an insignificant change in anxiety levels at the beginning and the end of Ramadan fasting. In a recent study conducted by Jandali et al. [28], they reported that mental health remained within its normal levels after Ramadan fasting. While our study and some previous studies, such as those by Alsowaid et al., 2021 and Furuncuoglu et al. [26,27], found no significant changes in mental health during Ramadan fasting, it is important to acknowledge that other studies have reported positive effects. For instance, Erdem [29] and Akan et al. [30] highlighted improvements in mental health, suggesting that the impact of fasting may vary depending on individual and contextual factors. Erdem et al. (2018), through their use of the Turkish version of the DASS (DASS-42), reported that Ramadan fasting has been very efficient in diminishing the levels of mental components, including depression, anxiety, and stress [29]. Akan et al. [30] documented, through their use of the Brief Symptom Inventory (BSI) and the Scale of Dimensions of Interpersonal Relationships (SDIR) on a sample of 40 healthcare professionals, that Ramadan fasting affected mental health through psychoneuroendocrine-mediated mechanisms. In a study of diabetic patients, Yousuf et al., 2021 found that after Ramadan fasting, a significant improvement was shown in the levels of depression, anxiety, and stress among the fasting group [31]. In the same context, Koushali et al., 2013 examined the effect of Ramadan fasting on emotional reactions among 313 nurses and reported that Ramadan fasting was effective at diminishing the levels of depression and stress [32]. In a recent randomized controlled trial (RCT) examined by Lauche et al., 2024 on mental well-being among participants, they suggested that Ramadan fasting with pre-Ramadan dietary and lifestyle advice was effectively associated with short-term enchantments in mental and physical well-being [33]. In a review based on the analysis of the results of eleven researchers, it was found that fasting affected all aspects of the mental health of students through feeling less depressed and anxious [1]. In another systematic review of 22 related papers regarding Ramadan fasting and mental health, it has been reported that the effects of Ramadan fasting on mental well-being were mixed, with minor positive and negative effects [34]. Surprisingly, a recent study documented that there was a significant reduction in the levels of glial-cell-derived neurotrophic factor (GDNF) between the beginning of Ramadan and the baseline [34]. GDNF is crucial for mental health through its role in the development, maintenance, and survival of different neurons [35]. Its levels were shown to be significantly reduced in depression, and this result indicated that Ramadan fasting might alter different biological mediators related to mental health [36].

The second axis of this research focused on the potential impact of Ramadan fasting on sleep quality and its components. It is well-known that sleep is a fundamental human necessity, a pivotal factor for both mental and physical rejuvenation that acts as the cornerstone of a well-rounded existence, and it is one of the most important physiological requirements [37].

Our study revealed a significant deterioration in sleep quality, particularly in subjective sleep quality, sleep duration, and sleep disturbance. This decline is consistent with findings from Faris et al. [38] and Al Harbi et al. [39], who also reported reduced total sleep time and increased sleepiness during Ramadan. These changes may be attributed to altered sleep schedules, increased nightly prayers, and environmental factors, such as delayed work hours and extended TV viewing. These results were confirmed by a linear mixed-effects model, which revealed that sleep quality deteriorated with Ramadan fasting after adjusting for all variables included in the models. An interesting indicator of this result was elucidated in this study through a significant increase in the number of participants that shifted from moderate and very active levels of physical activity to sedentary and light levels of physical activity.

By going back to the first axis, which indicated that Ramadan fasting was associated with normal mental health among participants, it was anticipated that they practiced good sleep. In contrast, poor sleep quality was the outcome during Ramadan fasting. The opposite impact of Ramadan fasting on mental health and sleep quality is justified by an improvement in the commitment of participants to ritual practices, and this might counterbalance the impact of poor sleep quality. The disruption in sleep quality could be explained by remarkable challenges, including changes in sleep schedules, onerous work routines, longer nightly prayers (praying Qiyam), and altered dietary habits during Ramadan [28,40,41]. Environmental factors, such as delayed work hours for shopping malls and restaurants and watching interesting TV programs until late at night, could also play a role in worsening sleep duration [42]. In addition, the delay in the starting hour of work during the holy month of Ramadan encourages bedtime and wake-up time and plays a role in reducing the total sleep time [11]. Another explanation for disruption in sleep quality may be explained by reductions in the circadian pattern of melatonin secretion, which affects sleep onset latency [43]. It is known that melatonin secretion helps in reducing sleep onset latency, thereby increasing total sleep duration and total sleep quality [44]. Until our research, published studies examined the impact of Ramadan fasting on sleep quality among different populations. [38], in their review of 24 studies, reported a reduction in the total sleep time (TST) from 7.2 h/night to 6.4 h/night before and during Ramadan, respectively. They also documented an increase in the score of the Epworth sleepiness scale (ESS) from 6.1 to 7.0 before and during Ramadan, respectively. In a recent study conducted by Akdeniz et al., 2024 on 73 male athletes, they found that the modifications in eating and sleeping patterns during the holy month of Ramadan can disrupt the sleep quality of athletes [45]. They also reported notable discrepancies in the subcomponents of daytime dysfunction and subjective sleep quality when investigating the impact of modifying mealtimes on training efficacy [38]. In a cross-sectional study that used the sleep quality scale (SQS) to assess sleep quality among the adult Saudi population during Ramadan, it was documented that poor sleep quality was common among the Saudi population [39]. Similarly, in another cross-sectional study conducted in Saudi Arabia on 386 medical students, it was reported that 75.1% of the participants had a reduction in their sleeping hours [46]. In another Arab country, Tunisia, an improvement in the scores of sleep quality, daytime dysfunction, and sleep disturbance with a reduction in the score of sleep efficiency was reported during fasting during the holy month of Ramadan [47]. In a study conducted on students from two universities in Turkey, the subcomponents of PSQI were changed as follows: deterioration in sleep latency, daytime dysfunction, sleep disturbances, subjective sleep quality. Meanwhile, there were no significant changes in the rest of the subcomponents of PSQI, including sleep efficiency, use of sleep medication, and sleep duration [48].

The third axis of this study was about body composition changes due to this religious ritual of fasting. The objectives of this study were to identify the impact of Ramadan fasting on different anthropometric and body composition measurements. The results of this study, which were confirmed by the linear mixed-effects model, revealed statistical reductions in all of the aforementioned measurements between that baseline and the end of Ramadan fasting, which was significant in all of the anthropometric and fat-related components and not significant in all water- and lean-related components except those measurements divided by body weight. Explanations of our study are clarified by investigations of a previous study [49]. The latter study reported that weight loss after Ramadan fasting was due to efficient utilization of body fat, reduction in beverage intake, reduction in glycogen-bound water stores, low volume of extracellular fluids due to reduced sodium intake, and moderate hypo-hydration, which is accompanied by some losses of body tissue. They also reported that the extent of dehydration and electrolyte imbalance during Ramadan fasting depends on the fasting season, physical fitness, and physical activity habits. Nikfarjad and Memari, 2016 reported that Ramadan fasting is characterized by changes in meal schedules and frequency and that total calorie intake significantly decreased [50]. They found that the loss of body weight might be due to a reduction in fluid intake, hypo-hydration, and little loss of body fat. Moreover, Rohin et al., 2013 suggested that during fasting, the weight loss could be due to the depletion of body fluids and not due to a reduction in body fat [51]. Norouzy et al., 2013 conducted a prospective observational study on a group of healthy adults, and their findings were in agreement with our study results [52]. They found a significant reduction among all males and females aged ≤35 years after Ramadan fasting in body weight (−2.2; −1.4), BMI (−2.1; −1.5), waist circumference (−1.3; −1.3), hip circumference (−1.9; −1.0), FM (−4.3; −2.3), and percent body fat (−2.5; −1.1). Similarly, Fernando et al., 2019 confirmed in their systematic review and meta-analysis the significant reductions in body weight and all parameters of body composition [53]. In the same context, a study conducted in Qatar reported that Ramadan fasting was associated with significant reductions in weight, BMI, FM, and FFM and insignificant reductions in muscle mass, waist circumference, and hip circumference [54]. In another Arab country, Bahrain, Jahrami et al., 2021 reported that between the baseline and the end of the holy month of Ramadan, there were significant reductions in weight (−1.87 kg); BMI (−0.69 kg/m^2^); body fat (0.87%); body surface area (0.03 m^2^); and lean mass (0.77 kg) [42]. In addition, in a longitudinal study conducted by López-Bueno et al., 2015 among sixty-two Berber Muslim females in Spain, they documented significant reductions among those females after Ramadan fasting in body weight, body fat percentage, and hip circumference, with significant values of *p* = 0.000, *p* = 0.008, and *p* = 0.000, respectively [55]. Furthermore, Al-Jafar et al., 2023 conducted a remarkable observational study and summarized that immediately after Ramadan fasting, significant reductions were noted in the means of weight (−1.6 kg); waist circumference (−1.95 cm); hip circumference (−2.86 cm); BMI (−0.60 kg/m^2^); and FM (−1.24 kg) [56]. In one of the studies that went beyond the benefits of Ramadan fasting on anthropometric and body composition measurements, Shehab et al., 2012 emphasized that the positive changes in measurements were associated with a progressive and significant increase in HDL-C and a significant decrease in LDL-C levels [57], and these results confirmed the crucial role of Ramadan fasting in lipid and lipoprotein control. In contrast to our study results, Urooj et al., 2020 found that Ramadan fasting did bring some alterations in body composition, with moderate alterations in intracellular and ECW content after fasting [58]. Similarly, in a recent study conducted in Saudi Arabia among young female university students, it was documented that no significant changes were noted in the anthropometric and body composition parameters after Ramadan fasting [59]. Actually, it is suggested that some of the socioeconomic and cultural variations between Muslim countries and communities may affect dietary practices and daily habits and that these differences may be responsible for such inconsistency and controversial findings across different studies.

## 5. Practical Implications and Considerations

The findings of this study provide important insights into health management during Ramadan fasting, particularly for university students. The significant decline in sleep quality observed among participants suggests a potential increase in fatigue and reduced cognitive function, which could negatively affect academic performance and overall well-being. To help mitigate these effects, students may benefit from adopting structured sleep hygiene practices, such as maintaining a consistent sleep schedule, reducing screen exposure before bedtime, and moderating caffeine intake in the evening.

Additionally, the reduction in physical activity levels highlights the need for strategies to encourage movement during Ramadan. Incorporating light to moderate activities, such as stretching, walking before iftar, or engaging in low-impact exercises, may help counteract the effects of prolonged sedentary behavior and support metabolic health. Given that physical inactivity during Ramadan may have long-term consequences, interventions that promote sustainable activity levels should be explored.

Although the observed reductions in body composition parameters, such as BMI and FM, were statistically significant, their small effect sizes suggest that these changes may not have substantial clinical relevance for all individuals. This underscores the need for personalized fasting strategies that take into account dietary intake, hydration, and activity levels to optimize overall health outcomes.

Future research should focus on developing targeted interventions to minimize sleep disturbances and maintain physical activity levels throughout Ramadan, ensuring both physical and mental well-being. Furthermore, as this study did not include direct assessments of physical fitness, future investigations should incorporate objective fitness measures to gain a more comprehensive understanding of the physiological impact of fasting.

## 6. Study Strengths and Limitations

This study offers a comprehensive look at how Ramadan fasting influences mental health, sleep quality, and body composition, areas that are often studied separately. By taking a holistic approach, it provides a clearer understanding of the overall impact of fasting in real-life settings. One of the study’s biggest strengths is its quasi-experimental pre–post design, allowing for direct comparisons among the same individuals. This reduces variability and makes the findings more reliable and meaningful. Another strong aspect is the use of validated tools to assess mental health (DASS-21), sleep quality (PSQI), and physical activity (GPPAQ), ensuring that the data are credible and comparable to previous research. Beyond BMI, the study employs bioelectrical impedance analysis (BIA) to provide a detailed breakdown of body composition, including FM and visceral fat, offering a more precise and insightful perspective. Statistical analysis further strengthens the study by adjusting for key confounders, such as age, sex, and physical activity, ensuring that the observed effects are genuinely due to fasting. The use of linear mixed-effects models also helps account for individual variations over time. Relevance to real-life settings is another highlight, particularly given that the study focuses on university students who must balance fasting with academic responsibilities. This makes the findings especially valuable for young fasting individuals, providing practical insights into how fasting affects their daily lives.

However, one key limitation is that dietary intake was not controlled. Because food choices and calorie intake vary during Ramadan, they could have influenced the observed changes in body composition. Future studies should incorporate dietary tracking methods, such as 24 h recalls or food frequency questionnaires, to provide a clearer picture of how food intake affects fasting outcomes. Another challenge is that physical activity and sleep quality were self-reported, which may introduce recall bias or subjectivity. People often misjudge their activity levels and sleep patterns, so using wearable fitness devices or actigraphy in future research would improve accuracy. The study’s duration is another factor to consider, as it only covers one Ramadan cycle (one month). While this provides valuable short-term insights, it does not clarify whether these effects persist, diminish, or accumulate over multiple years. A longitudinal study tracking participants over several fasting periods would help answer this question. Additionally, the sample size and generalizability should be noted. With only 77 university students from Jordan, the findings may not fully apply to older adults, individuals with pre-existing conditions, or those from different cultural and socioeconomic backgrounds. Expanding the sample size in future studies would improve the applicability of the results. Another limitation is the absence of a control group. Without a non-fasting comparison group, it is difficult to determine whether the observed changes were directly due to Ramadan fasting or simply seasonal or lifestyle variations. Future research should include both fasting and non-fasting groups to isolate the true effects of Ramadan fasting. Additionally, direct physical fitness measurements were not included. While the study assessed physical activity levels, it did not measure specific fitness indicators like VO_2_ max, endurance, or muscle strength. Adding these measurements in future research would provide a more complete understanding of how fasting impacts physical performance. Finally, statistical reporting could be enhanced by including confidence intervals (CIs) for key findings. While the study presents strong statistical results, adding 95% CIs would provide a clearer measure of the precision of the estimates. Despite these limitations, this study presents a well-rounded and insightful analysis of how Ramadan fasting affects mental health, sleep, and body composition. It highlights both the benefits, such as improvements in body composition, and the challenges, including sleep disturbances and reduced physical activity. Moving forward, future research should build on these findings by conducting longer-term studies, incorporating objective activity and sleep tracking, accounting for dietary intake, and expanding sample diversity. These improvements would help develop more effective health strategies to support individuals fasting during Ramadan while maintaining their well-being and daily performance.

## 7. Conclusions and Future Research

This study aimed to assess the impact of Ramadan fasting on mental health, sleep quality, and body composition among university students. In conclusion, the current findings indicate that there were no significant changes in the levels of depression, anxiety, or stress before and after Ramadan fasting, suggesting that fasting did not have a substantial effect on mental health in this population. However, sleep quality showed a significant decline, particularly in subjective sleep quality and duration, highlighting the need for strategies to improve sleep hygiene during Ramadan. Additionally, significant reductions in body composition parameters, such as BMI and waist circumference, were observed, though with small effect sizes, suggesting that while fasting may contribute to short-term changes in body composition, these may not be clinically significant for all individuals. These findings highlight the complex interplay between fasting, sleep, and physical health, underscoring the need for targeted interventions to mitigate negative effects, particularly on sleep quality. However, there is no statistical significance, despite a slight increase in depression and anxiety rates. Furthermore, our results showed a significant shift towards more sedentary and light activities during Ramadan. While Ramadan fasting was associated with positive changes in body composition, it also led to significant declines in sleep quality and a marked reduction in physical activity levels. These findings underscore the potential trade-offs of fasting, where physiological benefits may come at the cost of disrupted sleep patterns and increased sedentary behavior. Such disruptions could affect cognitive function, mood regulation, and overall well-being, particularly in students balancing fasting with academic responsibilities. Body composition scores also showed that Ramadan fasting resulted in a reduction in adiposity measures, such as weight, BMI, and waist circumference, and fat-related measures, such as the FM index and VFA. The results of the analysis of the relationship between these variables show that Ramadan fasting was significantly associated with changes in body composition in students after the adjustment of many variables (age, sex, and physical activity). Future research should aim to explore the long-term effects of Ramadan fasting through longitudinal studies that track physiological and behavioral changes over multiple years. Additionally, intervention-based studies examining sleep hygiene strategies and exercise programs tailored for fasting individuals could help mitigate the negative impacts of fasting on sleep and activity levels. Given the cultural and demographic differences in fasting practices, cross-cultural studies comparing the effects of Ramadan fasting across diverse populations would provide a more comprehensive understanding of its impact on health and well-being. Also, because Ramadan fasting is associated with a change in dietary intake and eating practices, we did not adjust for caloric intake. We recommend that future studies examine dietary intake and dietary habits when assessing the impact of Ramadan fasting on body composition, physical activity, and mental health, because diet could confound the association between Ramadan fasting and health outcomes and measures.

## Figures and Tables

**Table 1:** Demographic characteristics of study participants (n = 77).

Characteristics	n (%)
Sex	
Female	64 (72.73)
Males	24 (27.27)
Marital status	
Single	83 (94.32)
Married	5 (5.68)
Smoking	
No	53 (60.23)
Yes	27 (30.68)
Ex-smoker	8 (9.09)
Residence	
With my family	69 (78.41)
Students’ housing	5 (5.68)
Alone	14 (15.91)

**Table 2:** Change in depression, anxiety, and stress before and after Ramadan Fasting (N = 77).

	Before (T1)	After (T2)	Diff (T1–T2)	Effect Size	*p*-Value ^a^
DASS 21	15.90 ± 12.32	16.70 ± 13.94	−0.81 ± 8.31	−0.07 ± 1.00	0.4080
Depression	10.84 ± 8.14	11.23 ± 9.44	−0.39 ± 12.37	−0.05 ± 1.02	0.4769
Anxiety	11.05 ± 8.91	11.90 ± 9.93	−0.84 ± 8.92	−0.09 ± 1.00	0.3058
Stress	9.90 ± 7.97	10.27 ± 8.97	−0.38 ± 7.97	−0.05 ± 1.00	0.6161

Data expressed as mean and standard deviation were reported. ^a^ Significance level using the Wilcoxon test (also known as the Wilcoxon signed-rank test).

**Table 3:** Change in physical activity and sleep before and during Ramadan Fasting (N = 77).

	Before (T1)	After (T2)	Diff (T1–T2)	Effect Size	*p*-Value ^a^
Physical activity—2 levels					
Sedentary and light activity, %	3 (3.41)	55 (62.50)	N/A	N/A	<0.0001
Moderate and very active, %	85 (96.59)	33 (37.50)	N/A	N/A	
PSQI	5.73 ± 2.40	6.65 ± 3.08	−4.55 ± 8.18	−1.89 ± 3.4	<0.0001
Component 1: Subjective sleep quality	1.10 ± 0.79	1.52 ± 1.05	−0.42 ± 1.09	−0.53 ± 1.39	0.0009
Component 2: Sleep latency	1.14 ± 0.96	1.01 ± 0.97	0.13 ± 0.98	0.14 ± 1.02	0.2802
Component 3: Sleep duration	0.81 ± 0.89	1.47 ± 1.02	−0.66 ± 1.05	−0.74 ± 1.18	<0.0001
Component 4: Sleep efficiency	0.13 ± 0.34	0.16 ± 0.46	−0.03 ± 0.56	−0.08 ± 1.66	0.6328
Component 5: Sleep disturbance	1.21 ± 0.50	1.04 ± 0.55	0.17 ± 0.66	0.34 ± 1.33	0.0250
Component 6: Use of sleep medication	0.31 ± 0.69	0.26 ± 0.57	0.05 ± 0.76	0.07 ± 1.1	0.6349
Component 7: Daytime dysfunction	1.03 ± 0.71	1.19 ± 0.92	−0.17 ± 0.78	−0.24 ± 1.11	0.0719

Data expressed as mean ± standard deviation or frequency (percentage) were reported for continuous and categorical variables, respectively. ^a^ Significance level using the Wilcoxon test (also known as the Wilcoxon signed-rank test) for continuous variables and McNemer’s test for binary variables.

**Table 4:** Change in body composition before and after Ramadan fasting (N = 72).

	Before (T1)	After (T2)	Diff (T1–T2)	Effect Size	*p*-Value ^a^
Anthropometric measures					
Weight, kg	66.55 ± 15	65.2 ± 14.78	1.35 ±2.62	0.09 ± 0.17	<0.0001
Body mass index, Kg/m^2^	25.31 ± 4.84	24.8 ± 4.82	0.52 ± 0.96	0.11 ± 0.2	<0.0001
Waist circumference	87.92 ± 12.92	85.83 ± 12.87	2.08 ± 3.32	0.16 ± 0.26	<0.0001
Waist–hip ratio	0.9 ± 0.07	0.89 ± 0.07	0.02 ± 0.03	0.25 ± 0.41	<0.0001
Hip circumference	96.84 ± 7.93	96.24 ± 7.91	0.61 ± 1.53	0.08 ± 0.19	<0.0001
Water-related components					
Total body water	31.65 ± 7.23	31.48 ± 7.08	0.17 ± 0.95	0.02 ± 0.13	0.0703
Intracellular water	19.65 ± 4.64	19.56 ± 4.56	0.09 ± 0.58	0.02 ± 0.12	0.0634
Extracellular water	12 ± 2.6	11.92 ± 2.53	0.07 ± 0.4	0.03 ± 0.15	0.1242
Total body water by weight	47.94 ± 5.98	48.72 ± 6.17	−0.78 ± 2.34	−0.13 ± 0.39	0.0002
Fat-related components					
Fat mass index	8.95 ± 3.39	8.52 ± 3.4	0.42 ± 1.1	0.12 ± 0.32	<0.0001
Percent body fat	34.5 ± 8.13	33.44 ± 8.38	1.06 ± 3.2	0.13 ± 0.39	0.0002
Visceral fat area	113.7 ± 48.28	106.17 ± 48.47	7.54 ± 18.66	0.16 ± 0.39	<0.0001
Obesity degree	119.13 ± 21.97	116.74 ± 21.94	2.39 ± 4.45	0.11 ± 0.2	<0.0001
Body adiposity index	29.16 ± 4.39	28.87 ± 4.41	0.29 ± 0.92	0.07 ± 0.21	<0.0001
Skeletal and lean-related components					
Soft lean mass	40.67 ± 9.35	40.46 ± 9.17	0.21 ± 1.21	0.02 ± 0.13	0.0859
Fat-free mass index	16.36 ± 2.57	16.29 ± 2.55	0.07 ± 0.49	0.03 ± 0.19	0.0692
Skeletal muscle mass	23.63 ± 6.06	23.49 ± 5.95	0.14 ± 0.75	0.02 ± 0.12	0.0596
Skeletal muscle index	6.57 ± 1.13	6.56 ± 1.12	0.01 ± 0.17	0 ± 0.15	0.7240
Skeletal muscle mass by weight	35.63 ± 4.88	36.2 ± 5.03	−0.57 ± 1.84	−0.12 ± 0.38	0.0018
Skeletal muscle mass–visceral fat area ratio	0.26 ± 0.18	0.28 ± 0.2	−0.02 ± 0.06	−0.14 ± 0.32	<0.0001

Data expressed as mean ± standard deviation was reported. ^a^ Significance level using the Wilcoxon test (also known as the Wilcoxon signed-rank test).

**Table 5:** Associations between depression, anxiety, stress, sleep quality, and body compositions and Ramadan fasting using linear mixed-effect models.

	Model 1		Model 2		Model 3		Model 4	
	β (SE)	*p*-Value	β (SE)	*p*-Value	β (SE)	*p*-Value	β (SE)	*p*-Value
Mental health								
DASS 21	0.58 (1.38)	0.6766	0.57 (1.38)	0.6785	0.50 (1.38)	0.7177	−0.24 (1.95)	0.9027
Sleep quality								
PSQI score	0.82 (0.32)	0.0133	0.81 (0.32)	0.0145	0.82 (0.32)	0.0134	0.92 (0.45)	0.0419
Body composition								
Anthropometric measures								
Weight, kg	−1.36 (0.31)	<0.0001	−1.36 (0.31)	<0.0001	−1.35 (0.31)	<0.0001	−1.20 (0.46)	0.0116
Body mass index, Kg/m^2^	−0.52 (0.11)	<0.0001	−0.52 (0.11)	<0.0001	−0.52 (0.11)	<0.0001	−0.55 (0.17)	0.0017
Waist circumference	−2.10 (0.39)	<0.0001	−2.10 (0.39)	<0.0001	−2.09 (0.39)	<0.0001	−1.79 (0.58)	0.0029
Waist–hip ratio	−0.017 (0.003)	<0.0001	−0.017 (0.003)	<0.0001	−0.017 (0.003)	<0.0001	−0.014 (0.005)	0.0041
Hip circumference	−0.61 (0.18)	0.0011	−0.61 (0.18)	0.0010	−0.61 (0.18)	0.0011	−0.66 (0.27)	0.0175
Water-related components								
Total body water	−0.17 (0.11)	0.1296	−0.17 (0.11)	0.1289	−0.17 (0.11)	0.1379	−0.07 (0.17)	0.6668
Intracellular water	−0.09 (0.07)	0.1694	−0.09 (0.07)	0.1686	−0.09 (0.07)	0.1785	−0.04 (0.10)	0.6895
Extracellular water	−0.08 (0.05)	0.1024	−0.08 (0.05)	0.1016	−0.07 (0.05)	0.1109	−0.03 (0.07)	0.6639
Total body water by weight	0.77 (0.27)	0.0061	0.77 (0.27)	0.0062	0.79 (0.27)	0.0048	0.80 (0.41)	0.0527
Fat-related components								
Fat mass index	−0.42 (0.13)	0.0015	−0.42 (0.13)	0.0015	−0.43 (0.13)	0.0014	−0.43 (0.19)	0.0279
Percent body fat	−1.05 (0.37)	0.0061	−1.05 (0.37)	0.0062	−1.08 (0.37)	0.0048	−1.08 (0.56)	0.0548
Visceral fat area	−7.56 (2.17)	0.0009	−7.57 (2.17)	0.0008	−7.60 (2.17)	0.0008	−6.86 (3.26)	0.0383
Obesity degree	−2.40 (0.52)	<0.0001	−2.40 (0.52)	<0.0001	−2.39 (0.52)	<0.0001	−2.48 (0.79)	0.0024
Body adiposity index	−0.29 (0.11)	0.0081	−0.29 (0.11)	0.0082	−0.29 (0.11)	0.0087	−0.45 (0.16)	0.0068
Skeletal and lean-related components								
Soft lean mass	−0.21 (0.14)	0.1395	−0.21 (0.14)	0.1387	−0.21 (0.14)	0.1481	−0.09 (0.21)	0.6910
Fat-free mass index	−0.08 (0.06)	0.1936	−0.08 (0.06)	0.1925	−0.07 (0.06)	0.2050	−0.10 (0.09)	0.2649
Skeletal muscle mass	−0.14 (0.09)	0.1126	−0.14 (0.09)	0.1119	−0.14 (0.09)	0.1190	−0.07 (0.13)	0.5956
Skeletal muscle index	−0.01 (0.02)	0.7599	−0.01 (0.02)	0.7577	−0.01 (0.02)	0.8016	−0.002 (0.030)	0.9534
Skeletal muscle mass by weight	0.56 (0.21)	0.0108	0.56 (0.21)	0.0109	0.58 (0.21)	0.0081	0.58 (0.32)	0.0727
Skeletal muscle mass–visceral fat area ratio	0.02 (0.01)	0.0005	0.02 (0.01)	0.0005	0.02 (0.01)	0.0003	0.02 (0.01)	0.0449

Model 1 is unadjusted. Model 2 is adjusted for age. Model 3 is adjusted for age and sex. Model 4 is adjusted for age, sex, and physical activity.

## Data Availability

The data presented in this study are available upon request from the corresponding author due to privacy, legal, or ethical reasons).

## Abbreviations

DASS-21	Depression Anxiety Stress Scales-21
PSQI	Pittsburgh Sleep Quality Index
GPPAQ	General Practice Physical Activity Questionnaire
BMI	Body mass index
BMR	Basal metabolic rate
FM	Fat mass
FFM	Fat-free mass
TBW	Total body water
ECW	Extracellular water
ICW	Intracellular water
VFA	Visceral fat area
